# Synergistic Effects of Lauryl Gallate and Tamoxifen on Human Breast Cancer Cell

**DOI:** 10.18502/ijph.v49i7.3586

**Published:** 2020-07

**Authors:** Keihan GHATREH SAMANI, Effat FARROKHI, Aliye TABATABAEE, Narges JALILIAN, Mahbube JAFARI

**Affiliations:** 1. Clinical Biochemistry Research Center, Basic Health Sciences Institute, Shahrekord University of Medical Sciences, Shahrekord, Iran; 2. Department of Molecular Medicine, School of Advanced Technologies, Shahrekord University of Medical Sciences, Shahrekord, Iran; 3. Cellular and Molecular Research Center, Basic Health Sciences Institute, Shahrekord University of Medical Sciences, Shahrekord, Iran

**Keywords:** Breast cancer, Lauryl gallate, Gene expression, Apoptosis, Tamoxifen

## Abstract

**Background:**

Tamoxifen (TAM) is widely used for adjuvant therapy in breast cancer patients. Tamoxifen therapy may lead to serious side effects. Anti-apoptotic substances in combination with chemotherapy drugs can result in additive or synergistic effects. Lauryl gallate (LG), a Gallic acid derivative, has been proven to inhibit tumor growth, without affecting normal cells. This study aimed to investigate the synergistic effect of TAM and LG in breast cancer cell line (MCF-7).

**Methods:**

In this experimental study, performed in ShahreKord University of Medical Science, Iran in 2017, the MCF-7 cells were treated by final concentrations of 10 μM TAM alone, and in combination with 200 μM of LG. We also used EX-527, as SIRT-1 inhibitor to examine the role of SIRT1 in cell apoptosis. *BCL-2* and *SIRT1* gene expression were measured by real-time PCR method, and cell apoptosis was investigated by flow cytometry.

**Results:**

Tamoxifen alone and in combination with LG decreased *BCL-2* expression 2.64±0.75 and 6.38±1.9 fold, respectively, after 48 h (*P*<0.05). *SIRT1* expression was increased 1.67±0.22 and 2.47±0.34 - fold by TAM alone and in combination with LG, respectively (*P*<0.05). TAM alone and in combination with LG increased the percentage of apoptotic cells 15.79±2.81 and 60.67±6.23 percent, respectively after 48 h (*P*<0.001).

**Conclusion:**

The combination of LG and TAM is more effective for induction of apoptosis of breast cancer cells, compared to individual use of each. Thus, our data pave the way for new therapeutic options for suppressing breast cancer growth.

## Introduction

Breast cancer is the most prevalent causes of cancer mortality, among the female population in developing countries (1). Tamoxifen (TAM) is an anti-estrogenic drug widely used for adjuvant therapy in breast cancer patients (2). In addition, TAM is also used to prevent breast cancer in healthy high-risk women (3). Long term TAM therapy may lead to serious side effects, such as cardiovascular events, hepatic injury, etc. (4, 5).

Hence, the use of herbal medicine with anti-cancer effects, in combination with low concentrations of TAM may help to reduce TAM side effects. Lauryl gallate (LG) is a Gallic acid derivative with antioxidant effects, used as a food additive (6). LG has been proven to protect human cells from oxidative damage and to inhibit tumor growth, without affecting normal cells (7). In addition, LG has also been reported to induce apoptosis and inhibit the proliferation of cancer cells (8, 9).

The combination therapy might be a significant advantage for cancer patients, to increase the response rate and reduce side effects of cancer therapeutics. Combination therapies, promoting the effectiveness of TAM, have been previously investigated in several studies, using compounds, such as caffeic acid phenethyl ester (10), green tea (11) and thymoquinone (12).

Concurrent administration of LG has additive or synergistic effects with TAM, widely has been used for the treatment of breast cancer. Accordingly, in this study we investigated the efficacy of TAM and LG in breast cancer cells, with regard to apoptosis in these cells.

## Materials and Methods

### Cell culture

In this experimental study, the human breast cancer cell lines MCF-7 were obtained from the Pasteur Institute of Iran in 2017. Cells were routinely maintained in RPMI medium 1640 (Sigma, USA), containing 10% fetal bovine serum (FBS; Gibco, USA), 100 U/ml penicillin and 100 mg/ml streptomycin (Sigma, USA). Cells were incubated in a humidified incubator, containing 5% CO2 at 37 °C. Cell growth and cell division were daily controlled.

The study protocol was approved by the Shahrekord University of Medical Science Ethics Committee (IR.SKUMS.REC.1395.98).

### Cell proliferation assay

The MTT assay was used to determine LG concentration, causing 50% cell death (IC50). Briefly, the cells were seeded at a density of 15×10 ^3^ in 96-well plates, and allowed to attach for 24 h, before incubation with drugs.

Cells were then exposed to different doses of LG alone and in combination with 10 μM final concentration of TAM (10), for 48 hours. For each sample and for every concentration three wells were loaded. Thereafter, 20 μl MTT (Sigma-Aldrich; 5 mg/ml) solution was added to each well. After 4h incubation at 37 °C, the medium was replaced with 100 μL of 0.1 N HCl/isopropanol, in each well. The optical density of each well was determined at 570 nm, using an ELISA plate reader (Awareness, USA).

### Treatment

The cells were seeded in a 6-well plate at a density of 1×10 ^5^ cells per well. After 24 h, they were treated by 10 μM final concentration of TAM alone and in combination with 200 μM final concentration of LG, for 24 and 48 hours. Another group was also considered with TAM and LG, in combination with 2 μM final concentration of EX-527, as SIRT-1 inhibitor. A group was considered as control group cultured without any drug treatment. We used EX-527 to examine the role of SIRT1 in cell apoptosis. By this, we determined the contribution and role of each gene (*BCL-2* and *SIRT-1*) in the apoptosis induction.

### RNA isolation and cDNA synthesis

Total RNA was isolated from the cells, using Trizol (Invitrogen, California), according to the manufacturer’s instructions. RNA was quantified, using a Nanodrop 2000 C spectrophotometer (Thermo Scientific-USA). Then cDNA was synthesized from 0.4 mg total RNA, using random primer and the cDNA synthesis kit (Thermo Fisher Scientific-Waltham, MA).

### Real-Time Polymerase Chain Reaction (real-time PCR)

*BCL-2* and *SIRT-1* gene Expression were measured by quantitative real-time PCR. The experiments were performed, using Rotor-Gene 3000 real-time DNA amplification system (Corbett Research, Australia), and SYBR Green Method. Primers used for real-time PCR are listed in Table 1. Experiments were performed in triplicate, using 5 μL SYBR Green PCR Master Mix, 0.2 μL primer sets (10 μMol), 40 ng cDNA, and 3.6 μL nuclease-free H2O, in a final volume of 10 μL. The amplification was carried out under the following conditions: initial denaturing at 95 °C for 10 min, then 40 cycles of 95 °C for 15 sec, 59 °C for 20 sec and 72 °C for 25 sec. Quantitation of data was performed, using the comparative expression CT (ΔΔCT) method. GAPDH was used as a housekeeping gene. The gel electrophoresis of PCR product and melting curve analysis were investigated to ensure the specificity of the reactions.

**Table 1:** Primer sequences and product length

*** Genes ***	*** Primer sequences (5'–3') ***	*** Amplicon length (bp) ***	*** GeneBank reference ***
* BCL-2 *	Forward: GTGCTGAAGATTGATGGGATCG	118	NM_000633.2
	Reverse: TCAGTCTACTTCCTCTGTGATGTTG		
* SIRT-1 *	Forward: TGCTGGCCTAATAGAGTGGCA	102	NM_012238.4
	Reverse: CTCAGCGCCATGGAAAATGT		
* GAPDH *	Forward: ACACCCACTCCTCCACCTTTG	112	NM-002046.5
	Reverse: TCCACCACCCTGTTGCTGTAG		

### Flow cytometric analysis of cell apoptosis

The apoptosis kit (FITC Annexin V Apoptosis Detection Kit I, BD Biosciences, USA) was used to detect the apoptotic cells, according to the manufacturer’s recommendation. Briefly, MCF-7 cells were seeded in the 6-well plates (1 × 10 ^5^ cells per well) and incubated for 24 hours. Then they were treated similar to those previously mentioned. After 24 h and 48 h, the cells were trypsinized and washed in PBS. Overall, 100 μL of cells were transferred to a tube, 5 μL of FITC-conjugated Annexin V (Annexin V-FITC) and 5 μL of propidium iodide (PI) were added, followed by incubation for 15 min at room temperature, in the dark. The cells were diluted by the binding buffer and directly analyzed, using flow cytometry (Partec GmbH, Munster, Germany).

### Statistical analysis

All experiments were performed in triplicate. Data are expressed as mean±SEM. Statistical analysis was performed, using nonparametric Kruskal-Wallis test. Differences between two groups were investigated by the Mann-Whitney test. *P*<0.05 was considered statistically significant.

## Results

**Fig. 1:**
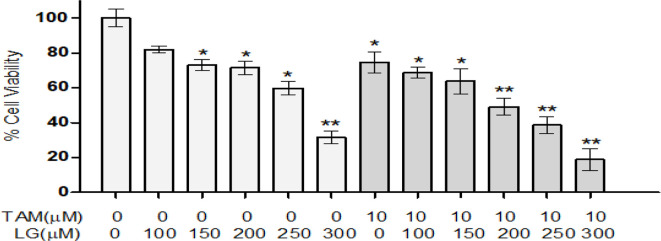
Cell viability in MCF-7 cell line. Effect of different doses of lauryl gallate (LG) alone and in combination with 10 μM tamoxifen (TAM) on cell viability, were determined by MTT assay for 48 hours. Data are presented as mean ± SEM of three independent experiments *P<0.05, **P<0.01

**Fig. 2:**
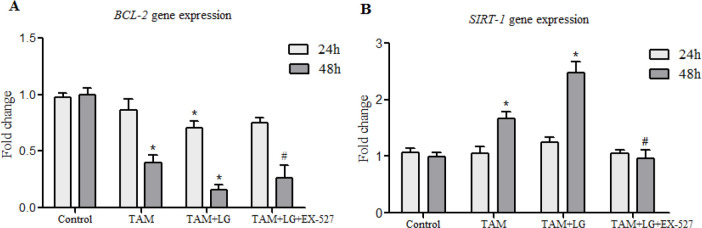
*BCL-2* and *SIRT1* gene expression in MCF-7 cells, after 24 and 48 hours TAM: Tamoxifen (10 μM), TAM+LG: Tamoxifen (10 μM) in combination with 200 μM of LG. TAM+LG+EX-527: Tamoxifen (10 μM) in combination with 200 μM of LG and 2 μM EX-527 Data are presented as mean ± SEM of three independent experiments. *Compared to the control group, # compared to TAM + LG group

Cell viability in MCF-7 cell line was determined by MTT assay (Fig. 1). The IC50 of the LG and in combination with 10 μM TAM was about 200 μmol/L. This concentration of LG was chosen for further experiments. We investigated the LG effects on *BCL-2* and *SIRT-1* expression in MCF-7 cells, in combination with TAM and EX-527(inhibitor of SIRT1), after 24 h and 48 hours. Our results showed TAM alone and in combination with 200 μM of LG significantly decreased *BCL-2* expression 2.64 ± 0.75 and 6.38±1.9–fold after 48 h, respectively, compared with the control group (*P*<0.001) (Fig. 2A). An increase in *BCL-2* expression was achieved when *SIRT1* was inhibited by EX-527 (*P*<0.05).

After 48h*, SIRT1* expression was significantly increased 1.67±0.22 and 2.47±0.34 fold by TAM alone and in combination with 200 μM of LG, respectively (*P*<0.05) (Fig. 2B). EX527 inhibited deacetylase activity of *SIRT1* in MCF-7 cell lines and thereby *SIRT1* expression was decreased 2.71±0.76 fold when SIRT1 was inhibited by EX-527 (*P*<0.05).

**Fig. 3:**
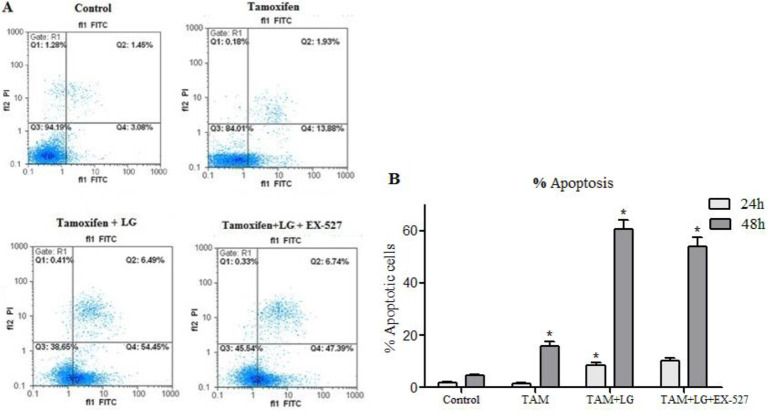
Flow cytometry analysis of Annexin V-FITC/PI stained apoptotic cells. A). Dot plot profile of cells after 48 h. B) Bar diagram of percent apoptotic cells after 24 and 48 hours TAM: Tamoxifen (10 μM), TAM+LG: Tamoxifen (10 μM) in combination with 200 μM of lauryl gallate. TAM+LG+EX-527: Tamoxifen (10 μM) in combination with 200 μM lauryl gallate and 2 μM EX-527 Data are presented as mean ± SEM of three independent experiments. *Compared to the control group, # compared to TAM + LG group

We further investigated the extent of apoptosis, by determining the percentage of Annexin V-stained cells. TAM alone and in combination with 200 μM of LG increased the percentage of apoptotic cells to 15.79±2.81% and 60.67±6.23%, respectively, after 48 h (Fig. 3) (*P*<0.001). On the other hand, the increase in apoptosis was reduced by EX-527, as *SIRT1* inhibitor (*P*<0.05). However, by *SIRT1* inhibition, the apoptosis rate was decreased only about 6%.

## Discussion

In this study, LG, as an antioxidant plays a role in reducing the growth of breast cancer cells. It does this by enhancing apoptosis through down-regulation of the anti-apoptotic gene expression, such as *BCL-2*. The complex biology of cancer development requires relatively complex treatment approaches. Thus, the application of synergistic combinations of drugs, play an important role in the treatment of cancer (13).

In this study, the combination of LG and TAM is more potent, compared to either agent alone, in preventing breast cancer cell growth. This prevention occurs through apoptotic effects of LG and TAM.

Anti-apoptotic substances in combination with chemotherapy drugs can result in additive or synergistic effects. For example, the combination of green tea and TAM have a synergistic effect on inhibiting the growth of cancer cell (11). Moreover, curcumin in combination with TAM may have therapeutic benefit for prevention of breast cancer cells growth (14).

LG is a Gallic acid derivative with antioxidant effects, used as a food additive. It may be possible to use lower doses of chemical drugs and minimize the associated side effects, by using concurrent intake of herbal medicine that has fewer side effects, compared to chemotherapy drugs.

The synergistic effects of other chemotherapy drugs with Gallic acid derivative have also been reported in a study (15). Dhima et al. have shown pre-treatment with epigallocatechin-3-gallate (EGCG) sensitised leiomyosarcoma cells to cisplatin (15). Overall, synergistic combinations of herbal medicines and chemotherapy drugs may use for enhancing efficacy, reducing side effects, immune modulation, and abrogating drug resistance (13).

SIRT1 is a member of the sirtuin family involved in genomic stability and cell survival, acting by deacetylation of histone and affecting some cell cycle regulators. There is a controversy about the role of sirtoin, as a tumor suppressor gene or as a proto-oncogene (16, 17).

In our study, TAM increased the *SIRT-1* gene expression, and LG intensified this effect. This effect was confirmed, by using EX527 as inhibiting the activity of *SIRT1*. But, by *SIRT1* inhibition, the apoptosis rate was decreased only about 6%. The synergistic effects of tamoxifen and lauryl gallate have a greater effect on the *BCL-2* than *SIRT1* gene at induction of apoptosis. But, it seems that the increase in TAM and LG -induced apoptosis, is partly due to the effect of *SIRT-1* on *BCL-2* expression. Kuo et al. showed that *SIRT1* suppresses breast cancer growth, through down-regulation of the *BCL-2* gene (18).

## Conclusion

Overall, compounds, such as LG strengthen the effects of drugs such as TAM, in inhibition of cancer cell growth. Further in vivo experiments needed, to confirm and validate these findings. Our data pave the way for using low-dose chemotherapy drugs and reducing the associated side effects of these drugs.

## Ethical considerations

Ethical issues (Including plagiarism, informed consent, misconduct, data fabrication and/or falsification, double publication and/or submission, redundancy, etc.) have been completely observed by the authors.
